# Retrospective analysis of different application methods in intratympanic glucocorticoid therapy for treatment of idiopathic SSNHL: A comparative outcome study

**DOI:** 10.1007/s00405-025-09432-7

**Published:** 2025-05-20

**Authors:** C. Schmit, F. Keller, T. Gottfried, M. Santer, A. Tröger, M. Kloppenburg, J. Klarer, A. Stenzl, J. Schmutzhard, B. Hofauer, D. Dejaco

**Affiliations:** 1 Universitätsklinik Für Hals-, Nasen- und Ohrenheilkunde, Medizinische Universität Innsbruck, Anichstraße 35, A-6020 Innsbruck, Österreich; 2 Univ.-Klinik Für Innere Medizin IV (Nephrologie Und Hypertensiologie), Medizinische Universität Innsbruck, Innsbruck, Austria

**Keywords:** Sudden hearing loss, Intratympanic glucocorticoid injection, CO2-laser, Hearing outcomes

## Abstract

**Purpose:**

Glucocorticoids for sudden sensorineural hearing loss (SSNHL) can be administered systemically or via intratympanic injection. This study evaluates the impact of different intratympanic injection techniques on patient outcomes.

**Methods:**

A retrospective analysis was conducted on patients diagnosed with idiopathic SSNHL (January 2008–May 2023). Patients were grouped by application technique: (1) transtympanic injection under local anesthesia (TI), (2) CO_2_ laser paracentesis under general anesthesia (LP), and (3) VT application under general anesthesia (VT). Primary outcomes included hearing improvement and tympanic membrane perforation closure time.

**Results:**

The study included 99 patients: 28 with TI, 34 with VT, and 37 with LP. Groups showed no significant differences in sex, affected side, therapy initiation interval, pretreatment type, preoperative BCPTA4, subjective improvement, or treatment-related pain (all *p* > 0.1). Hearing thresholds improved significantly in the LP and VT groups with large (d = 0.832, *p* < 0.001) and medium (d = 0.513, *p* = 0.01) effect sizes, respectively, but not in the TI group (d = 0.294, *p* = 0.137). Tympanic membrane closure time differed significantly among groups (*p* < 0.001).

**Conclusions:**

LP and VT significantly improved hearing thresholds in SSNHL patients. LP demonstrated the highest efficacy, consistent results across SSNHL severities, and the fastest tympanic membrane closure, making it the preferred method.

## Introduction

Sudden sensorineural hearing loss (SSNHL), traditionally defined as a sudden onset of sensorineural hearing loss of 30 dB or greater across at least three audiometric frequencies, is commonly encountered in otorhinolaryngology practice [1, 2]. While the identifiable causes of SSNHL are diverse, encompassing various infectious to vascular etiologies [1, 3–9], the majority of cases present an unknown etiology and are thus classified as idiopathic SSNHL [3].

Although SSNHL with identifiable causes can be subjected to specific therapeutic interventions, there remains considerable debate regarding the optimal treatment strategies for idiopathic SSNHL [1]. In alignment with leading hypotheses concerning the pathophysiology of idiopathic SSNHL, a broad array of therapeutic agents has been investigated [10]. Despite reports of a substantial spontaneous remission rate for idiopathic SSNHL [11], empirical treatment with glucocorticoids has gained widespread acceptance, albeit with inconsistent findings in the literature [10, 12–15].

Anti-inflammatory glucocorticoids can be administered systemically or locally via intratympanic cortisone injection (ICI) [1, 14]. Due to the lack of systemic absorption [16], ICIs are frequently used in patients with contraindications to systemic glucocorticoid therapy [1] or as a salvage treatment [17]. Additionally, their use in combination with systemic corticosteroids [18, 19] and as an equivalent alternative primary therapy has also been reported in literature [20–22].

In addition to the absence of systemic side effects, animal studies have demonstrated higher perilymph corticosteroid levels with intratympanic injections compared to intravenous administration [16]. Correspondingly a recent meta-analysis has shown superior outcomes in hearing threshold improvement and recovery rates in patients treated with primary ICI compared to systemic application [23].

Despite the multiple reported applications and the potential status as an alternative primary therapy, the use of intratympanic cortisone therapy for the treatment of idiopathic SSNHL remains non-standardized [14]. Currently, there are no recommendations regarding the specific substance used, the frequency of administration, the intervals between injections or the ICI technique [24]. Intratympanic steroid administration can be performed either through minimally invasive repeated injections under local anesthesia, myringotomy by CO2 laser or ventilation tube insertion, the latter both typically conducted under general anesthesia.

This study aims to evaluate whether application-specific differences affect the outcomes of patients suffering from an unilateral idiopathic SSNHL.

## Materials and methods

We retrospectively analyzed the medical records of adult patients diagnosed with idiopathic SSNHL, who were admitted to the Department of Otorhinolaryngology, Head and Neck Surgery at the Medical University of Innsbruck, Austria between January 2008 and May 2023. A total of 107 patients records were reviewed to ensure compliance with inclusion and exclusion criteria. The study adhered to the principles outlined in the Declaration of Helsinki and Good Clinical Practice guidelines.

### Inclusion criteria

The inclusion criteria were:

1. Diagnosis of unilateral idiopathic SSNHL.
2. Disease duration of less than 2 months.
3. Primary or salvage therapy involving intratympanic corticosteroid administration.

### Exclusion criteria

The exclusion criteria were:

1. Detection of an alternative cause for the unilateral hearing loss during the treatment course.
2. Discontinuation of the therapy regimen.
3. Absence of audiometric examination post-therapy.

### Study population

The study population consists of three treatment groups based on the method of intratympanic glucocorticoid administration. The following groups were defined in our retrospective analysis:

- Transtympanic injection under local anaesthesia (TI)

Patients in this group received microscopically targeted glucocorticoid (Dexabene^©^ 4 mg) injections into the middle ear under local anaesthesia. The treatment protocol consisted of intratympanic glucocorticoid injections administered on three consecutive days. Patients were informed about post-application care and monitored in an outpatient setting.

- Myringotomy by CO2 laser (LP)

Patients in this group underwent myringotomy with CO2 laser followed by targeted glucocorticoid (Dexabene^®^ 4 mg) application into the middle ear under general anesthesia. Subsequent glucocorticoid applications were performed without anesthesia. The treatment regimen involved intratympanic glucocorticoid application on three consecutive days. Patients were advised on post-application care and monitored accordingly.

- Insertion of a ventilation tube (VT)

Patients in this group had a ventilation tube inserted followed by targeted glucocorticoid (Dexabene^®^ 4 mg) application into the middle ear under general anesthesia. Subsequent glucocorticoid applications were performed without anesthesia. The therapy involved intratympanic glucocorticoid application on three consecutive days. Patients received instructions on post-application care and were monitored for an appropriate period.

### Audiometry examination

Audiometry was conducted by a trained audiologist. The Bone Conduction Pure Tone Average (BCPTA4) was calculated using hearing threshold at 500 Hz, 1 kHz, 2 kHz and 4 kHz. The resulting BCPTA4 were further divided into three equal tertiles based on the distribution of pre-therapeutic hearing thresholds, using the 33rd and 66 th percentiles as cut-off values. Based on the pre-therapeutic BCPTA4, propensity score matching was then performed in the main cohort as well as in the tertile subgroups.

### Assessment of outcome

The primary outcome measures were post-therapeutic hearing improvement and time to closure of the tympanic membrane perforation.

### Hearing improvement

Pre-, and post-therapeutic BCPTA4 were calculated after therapy completion. Hearing improvement was defined as the difference between pre-, and post-therapeutic BCPTA4.

### Time to closure

Follow-up otoscopic examinations were conducted at regular intervals to assess tympanic membrane perforation healing. The time of closure was documented for each patient, defined as the number of days from myringotomy to documented perforation closure. Follow-up continued until the ninetieth postoperative day.

### Subjective improvement

During follow-up examinations, patients were asked to subjectively assess their hearing improvement. Subjective improvement was documented for each patient.

### Pain assessment

Patients were queried about pain experienced during and after treatment at follow-up examinations. Reported pain was documented for each patient.

### Complications

The patient files were examined for possible follow-up interventions caused by the therapeutic intervention. Any necessary operative revision were documented.

### Statistical analysis

To describe continuous variables, the median and interquartile range (IQR) were used. For discrete variables, absolute and relative frequencies were presented. No missing values were observed in the patient characteristics.

Differences in continuous variables between groups were tested using the Kruskal–Wallis rank sum test. For discrete variables, the Chi-squared test and Fisher’s exact test were applied. Intra-patient differences in BCPTA4 within groups were tested using the Wilcoxon signed-rank test (paired).

To evaluate the effect size of treatment efficacy, specifically the intra-individual (paired) change in BCPTA4, Cohen’s d was calculated. The independence of BCPTA4 improvement and treatment was assessed using the Chi-squared test.

Absolute and relative BCPTA4 changes within treatment and BCPTA4 subgroups are displayed using boxplots. For the same groupings, relative frequencies of patients with an objective improvement are presented in a bar plot. Paired BCPTA4 changes within each patient are illustrated using a Swimmer arrow plot.

The cumulative incidences of perforation closure within treatments were estimated using Kaplan–Meier survival analysis. Differences in closure probability between treatments over time were tested using the log-rank test.

We allowed for a type 1 error or 5% with all hypotheses tested as two-sided. Analyses were carried out using R (version 4.4.2; R Foundation for Statistical Computing, Vienna, Austria) [25].

## Results

### Clinical characteristics in the entire study population

The study included 99 patients, of whom 30% were female and 70% were male, resulting in a female-to-male ratio of 1:2.3. SSNHL affected the right ear in 43% and the left ear in 57%. The median age of the study cohort was 59 years. The median interval between clinical presentation and the first intratympanic injection was 4 days.

Pretreatment with intravenously administered glucocorticoids was observed in 89%, while 11% were treated exclusively with orally administered glucocorticoids. Primary therapy using ICI was not conducted in any patient. Regarding the treatment groups, 28% received transtympanic instillation of glucocorticoids (TI), 34% underwent tube insertion (VT) followed by instillation, and 37% had laser paracentesis (LP) with subsequent glucocorticoid instillation.

The median preoperative BCPTA4 for the entire cohort was 58 dB. The VT group had a median preoperative BCPTA4 of 52 dB, the LP group 55 dB, and the TI group 68 dB (Table 1).

**Table 1:** Clinical characteristics in the entire study population

*Characteristic*	Overall (*N* = 99)	TI-Group (*N* = 28)	VT-Group (*N* = 34)	LP-Group (*N* = 37)
*Sex*				
* Male*	69 (70%)	18 (64%)	24 (71%)	27 (73%)
* Female*	30 (30%)	10 (36%)	10 (29%)	10 (27%)
*Side*				
* Right*	43 (43%)	8 (29%)	19 (56%)	16 (43%)
* Left*	56 (57%)	20 (71%)	15 (44%)	21 (57%)
*Age*^*1*^	59 (51, 73)	53 (49, 67)	62 (53, 73)	56 (51, 73)
*Pretreatment*				
* Oral*	11 (11%)	1 (4%)	5 (15%)	5 (14%)
* I.v*	88 (89%)	27 (96%)	29 (85%)	32 (86%)
*Preop. BCPTA4 (dB)*^*1*^	58 (39, 81)	68 (47, 88)	52 (39, 79)	55 (39, 80)

^1^Median (Q1, Q3)

### Clinical characteristics of the subgroups

The main study population was further divided into three equal tertiles based on the distribution of pre-therapeutic hearing thresholds, using the 33rd and 66 th percentiles as cut-off values (Table 2).

**Table 2:** Clinical characteristics in the equally distributed tertile subgroups

*Characteristic*	Overall (*N* = 99)	1 st Subgroup (*N* = 34)	2nd Subgroup (*N* = 33)	3rd Subgroup (*N* = 32)
*Sex*				
* Male*	69 (70%)	22 (65%)	29 (88%)	18 (56%)
* Female*	30 (30%)	12 (35%)	4 (12%)	14 (44%)
*Side*				
* Right*	43 (43%)	20 (59%)	14 (42%)	9 (28%)
* Left*	56 (57%)	14 (41%)	19 (58%)	23 (72%)
*Age *^*1*^	59 (51, 73)	52 (44, 60)	62 (52, 73)	67 (56, 78)
*Pretreatment*				
* Oral*	11 (11%)	3 (9%)	6 (18%)	2 (6%)
* I.v*	88 (89%)	31 (91%)	27 (82%)	30 (94%)
*Preop. BCPTA4 (dB)*^*1*^	58 (39, 81)	36 (26, 40)	58 (51, 69)	89 (83, 117)
*Type of surgery*				
* TI*	28 (28%)	7 (21%)	9 (27%)	12 (38%)
* VT*	34 (34%)	13 (38%)	12 (36%)	9 (28%)
* LP*	37 (37%)	14 (41%)	12 (36%)	11 (34%)

^1^Median (Q1, Q3)

#### 1^st^ Subgroup (BCPTA4 range [17.5, 45])

The first subgroup comprises patients with a preoperative BCPTA4 at or below the 33^rd^ percentile of the pretherapeutic hearing threshold distribution, ranging from 17.5 to 45 dB.

The median preoperative BCPTA4 value for the first subgroup was 36 dB.

The first subgroup consists of 34 patients of which 35% were female, while 65% were male. In 59% the right side was affected, while the left side was impacted in 41%. The median age of the first subgroup was 52 years. The median interval between clinical presentation and the first intratympanic injection was 4 days.

Pretreatment with intravenously administered glucocorticoids was observed in 91%, while 9% were treated exclusively with orally administered glucocorticoids. In the first subgroup 21% received transtympanic instillation of glucocorticoids (TI), 38% underwent tube insertion (VT) followed by instillation, and 41% had laser paracentesis (LP) with subsequent glucocorticoid instillation.

#### 2^nd^ Subgroup (BCPTA4 range [45, 76.2])

The second subgroup consists of patients with a preoperative BCPTA4 between the 34^th^ and 66^th^ percentile of the pretherapeutic hearing threshold distribution, ranging from 45 to 76.2 dB.

The median preoperative (BCPTA4) value for the second subgroup was 58 dB.

The second subgroup consists of 33 patients of which 12% were female, while 88% were male. The right side was affected in 42% while the left side was involved in 58%. The median age of the second subgroup was 62 years. The median interval between clinical presentation and the first intratympanic injection was 5 days.

Pretreatment with intravenously administered glucocorticoids was done in 82%, while 18% were treated exclusively with orally administered glucocorticoids. In the second subgroup 27% received transtympanic instillation of glucocorticoids (TI), 36% underwent tube insertion (VT) followed by instillation, and 36% had laser paracentesis (LP) with subsequent glucocorticoid instillation.

#### 3^rd^ Subgroup (BCPTA4 range [76.2, 125])

The third subgroup includes patients with a preoperative BCPTA4 above the 66^th^ percentile of the pretherapeutic hearing threshold distribution, ranging from 76.2 to 125 dB.

The median preoperative BCPTA4 value for the second subgroup was 89 dB.

The third subgroup consists of 32 patients of which 44% were female, while 56% were male. SSNHL affected the right ear in 28% and the left ear in 72%. The median age of the second subgroup was 67 years. The median interval between clinical presentation and the first intratympanic injection was 3 days.

Pretreatment with intravenously administered glucocorticoids was done in 94%, while 6% were treated exclusively with orally administered glucocorticoids. In the third subgroup 38% received transtympanic instillation of glucocorticoids (TI), 28% underwent tube insertion (VT) followed by instillation, and 34% had laser paracentesis (LP) with subsequent glucocorticoid instillation.

### Assessment of outcome parameters in the entire study population

Of the 99 patients, 49% reported subjective improvement in hearing during therapy. At 59% the subjective improvement was highest in the LP group and lowest in the TI group at 39%. Subjective improvement was reported in the VT group in 47%.

Pain during treatment was reported in 8.1%. With 12% the VT group had the highest incidence of pain, while the LP group had the lowest at 5.4%.

The median postoperative BCPTA4 in all patients was 45 dB representing a significant improvement over the preoperative BCPTA4 (*p* < 0.001). The TI group had a median postoperative BCPTA4 of 62 dB, the VT group of 43 dB and the LP group of 44 dB. The median absolute hearing threshold improvement was −2 dB in the TI group, −3 dB in the PR group and −8 dB. The median relative hearing threshold improvement was −5% in the TI group, −5% in the PR group and −23% in the LP group.

The median time to closure of tympanic membrane perforation was 36 days. The VT group had the longest median time to closure with 90 days, while the TI group had the shortest median time to closure with 19 days. The LP group had a median time to closure of 30 days.

In a total of 4%, revision surgery was necessary in the further course. All four cases belonged to the PR group. There were no complications in either the LP group or the TI group (Table 3).

**Table 3:** Outcome parameters in the entire study population

*Characteristic*	Overall (*N* = 99)	TI-Group (*N* = 28)	VT-Group (*N* = 34)	LP-Group (*N* = 37)
*Subj. Improvement*	49 (49%)	11 (39%)	16 (46%)	22 (59%)
*Pain*	8 (8%)	2 (7%)	4 (12%)	2 (5%)
*Postop. BCPTA4 (dB)*^*1*^	45 (29, 73)	62 (27, 91)	43 (29, 59)	44 (29, 59)
*Change in BCPTA4*^*1*^				
* Absolute (dB)*	−5 (−25, 3)	−2 (−26, 6)	−3 (−31, 4)	−8 (−20, −3)
* Relative (%)*	−11 (−39, 3)	−5 (−38, 8)	−5 (−47, 7)	−23 (−38, −6)
*Time to closure*^*1*^	36 (22, 90)	19 (11, 29)	90 (90, 90)	30 (22, 41)
*Revision Surgery*	4 (4%)	0 (0%)	4 (12%)	0 (0%)

^1^Median (Q1, Q3)

### Assessment of outcome parameters in the tertile subgroups

The main study population was further divided into three equally distributed subgroups based on the calculated pre-therapeutic BCPTA4 (Table 4)*.*

**Table 4:** Outcome parameters in the equally distributed tertile subgroups

*Characteristic*	Overall (*N* = 99)	1 st Subgroup (*N* = 34)	2nd Subgroup (*N* = 33)	3rd Subgroup (*N* = 32)
*Subj. Improvement*	49 (49%)	12 (35%)	18 (55%)	19 (59%)
*Pain*	8 (8%)	5 (15%)	1 (3%)	2 (6%)
*Postop. BCPTA4 (dB) *^*1*^	45 (29, 73)	33 (19, 44)	49 (38, 59)	78 (46, 95)
*Change in BCPTA4*^*1*^				
* Absolute (dB)*	−5 (−25, 3)	−3 (−8, 4)	−11 (−25, 3)	−25 (−46, 0)
* Relative (%)*	−11 (−39, 3)	−7 (−32, 8)	−18 (−38, 4)	−23 (−48, 0)
*Time to closure*^*1*^	36 (22, 90)	43 (22, 90)	35 (21, 90)	34 (26, 84)
*Revision Surgery*	4 (4%)	3 (9%)	0 (0%)	1 (3%)

^1^Median (Q1, Q3)

#### 1^st^ Subgroup

Of the 34 patients, 35% reported subjective improvement in hearing during therapy. Pain during treatment was reported by 15%. The median postoperative BCPTA4 for the first subgroup was 33 dB. The median absolute hearing threshold improvement in the first subgroup was −3 dB while the median relative hearing threshold improvement was −7%. The median time to closure of tympanic membrane perforation was 43 days.

#### 2^nd^ Subgroup

In the second subgroup a total of 55% reported subjective improvement in hearing during therapy. Pain during treatment was reported by 3%. The median postoperative BCPTA4 for the second subgroup was 49 dB. The median absolute hearing threshold improvement in the second subgroup was −11 dB while the median relative hearing threshold improvement was −18%. The median time to closure of tympanic membrane perforation was 35 days.

#### 3^rd^ Subgroup

Of the 32 patients, 59% reported subjective improvement in hearing during therapy. Pain during treatment was reported by 6.3%. The median postoperative BCPTA4 was 78 dB. The median absolute hearing threshold improvement was −25 dB while the median relative hearing threshold improvement was −23%. The median time to closure of tympanic membrane perforation was 34 days.

### Statistical evaluation of the main cohort

No significant differences between the three therapy modality groups in terms of sex, affected side, interval between first contact and initiated therapy, type of pretreatment, and preoperative BCPTA4 (all *p* > 0.1) have been noticed. Subjective improvement and pain during treatment also did not differ significantly between the groups (*p* = 0.3 and *p* = 0.7, respectively).

**Fig. 1:**
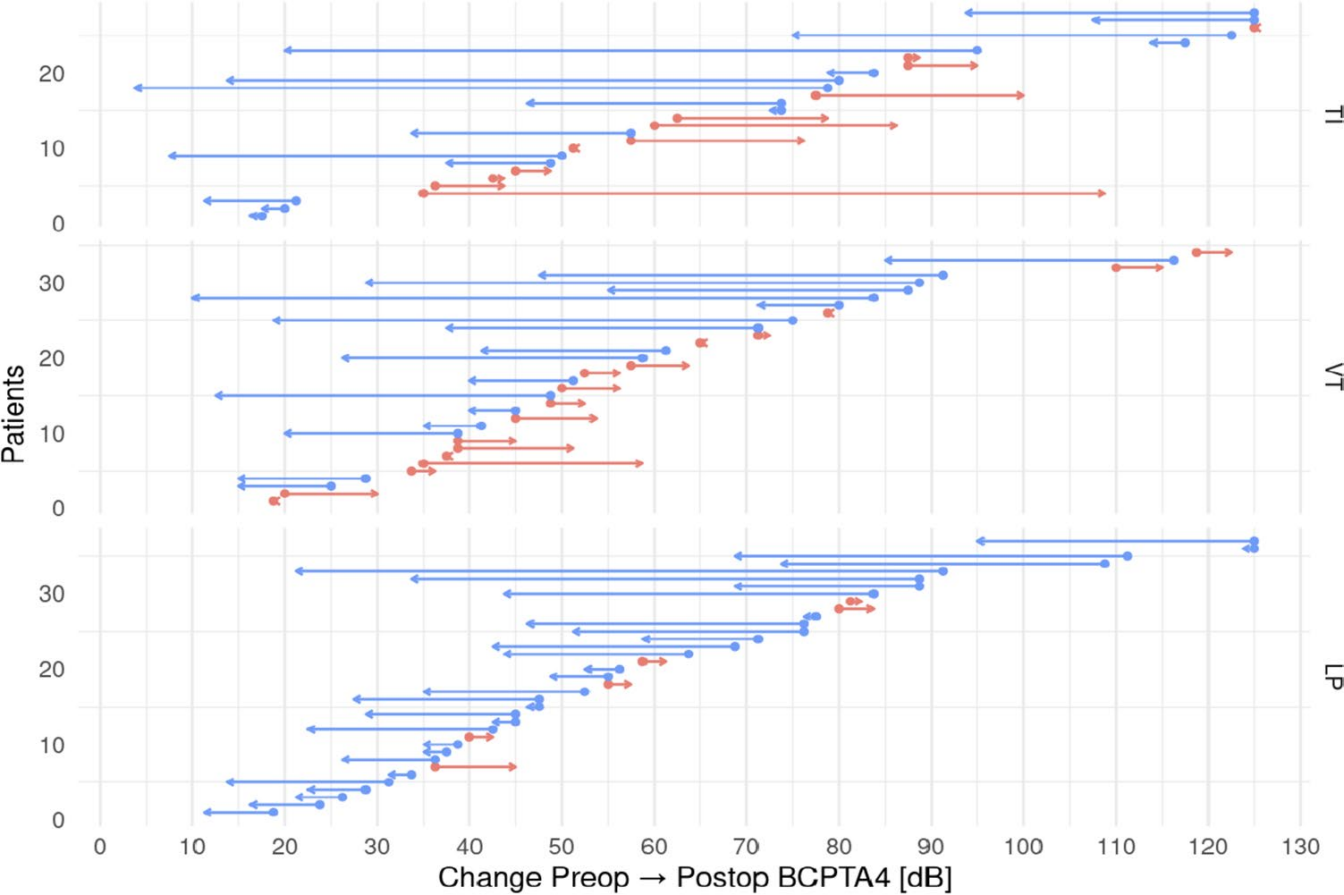
Change of BCPTA4 by treatment modality. Graphical representation of the measured changes in BCPTA4 in the entire study population. An improvement in the hearing threshold is shown in blue colouring, while a deterioration is shown in red colouring of the arrows

While preoperative and postoperative BCPTA4 did not differ significantly between the three therapy modality groups (all *p* > 0.3), and no significant differences in relative or absolute change in BCPTA4 was observed post-therapy, the LP group showed a significantly better objective improvement of the hearing threshold comparing to the other two treatment modalities (*p* = 0.007). In the LP and the PR group a significant change in hearing threshold with large and medium effect size was observed post-therapy (*p* < 0.001; d = 0.832 respectively *p* = 0.01; d = 0.513). No significant improvement in the hearing threshold post-therapy was found in the TI group (*p* = 0.294; d = 0.294) (Fig. 1).

**Fig. 2:**
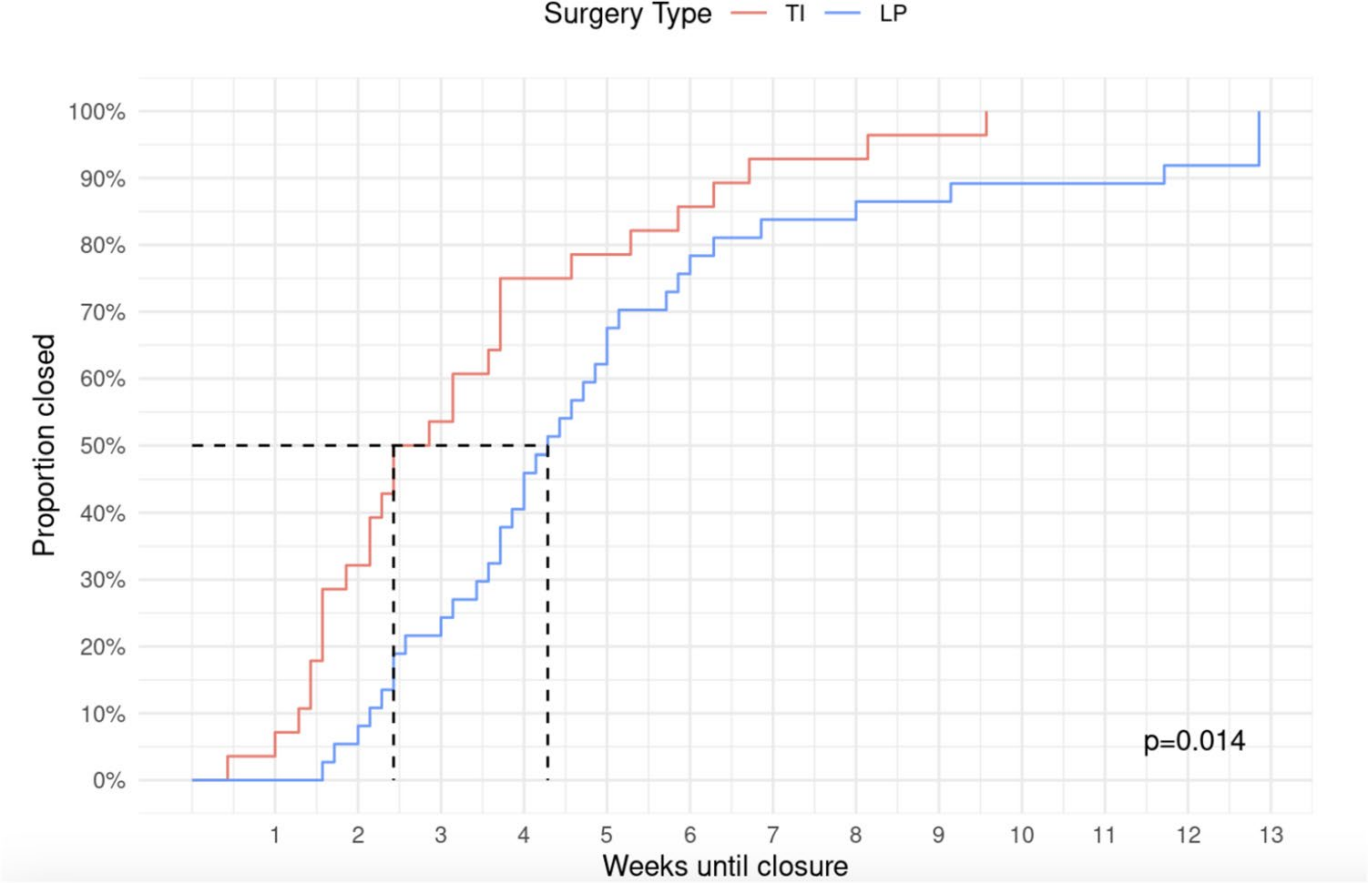
Time of closure probability by treatment modality. The dotted line shows a graphical representation of the median time of closure in patients treated by TI & LP

Regarding time to closure of tympanic membrane perforation, a significant difference was found among the three therapy modality groups (*p* < 0.001). The log rank test shows a significant difference in the probability of closure between the LP group and the TI group (Fig. 2).

With regard to the number of follow-up interventions required due to complications there was a significant difference between the respective treatment modalities (*p* = 0.018).

#### Statistical evaluation of the subgroups

Considering the three subgroups, no significant differences in terms of interval between first contact and initiated therapy or type of pretreatment have been found (*p* = 0.2). Subjective improvement and pain during treatment also did not differ significantly between the subgroups (*p* = 0.11 and *p* = 0.2, respectively). In accordance with the subdivision made, there was a significant difference in pre-, and posttherapeutic BCPTA4 between the three tertile subgroups. In addition, there were significant differences between the three subgroups with regard to age (*p* < 0.001), sex (*p* = 0.016) and the affected side (*p* = 0.042).

**Fig. 3:**
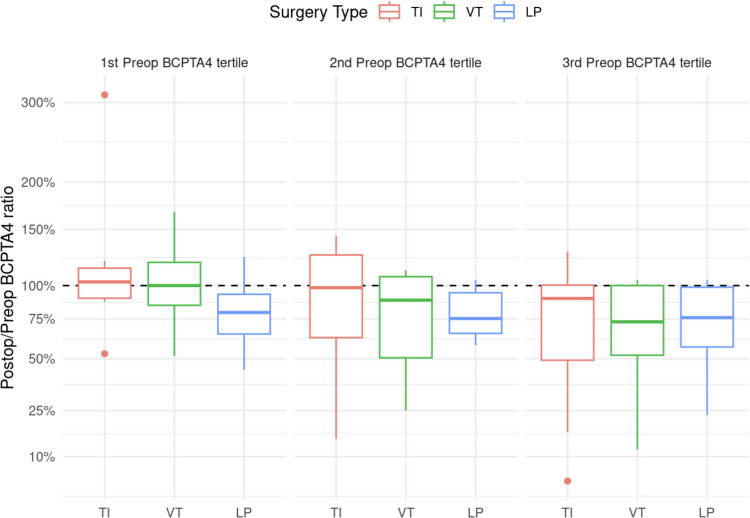
Relative Improvement in BCPTA4 by therapy modality and subgroup. Graphical representation of the relative improvement of the hearing threshold in all three subgroups, depending on the treatment modality used

**Fig. 4:**
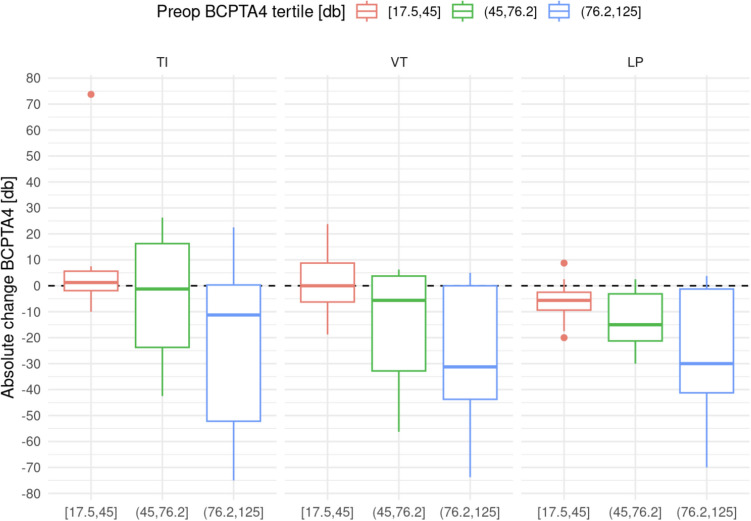
Absolute Improvement in BCPTA4 by therapy modality and subgroup. Boxplot of the absolute improvement of the hearing threshold in all three treatment modality groups split by subgroup

While the absolute difference in the BCPTA4 was statistically significant different from 0 (*p* = 0.003), there were no significant differences between the three subgroups with regard to the relative difference (*p* = 0.14) (Figs. 3 and 4).

**Fig. 5:**
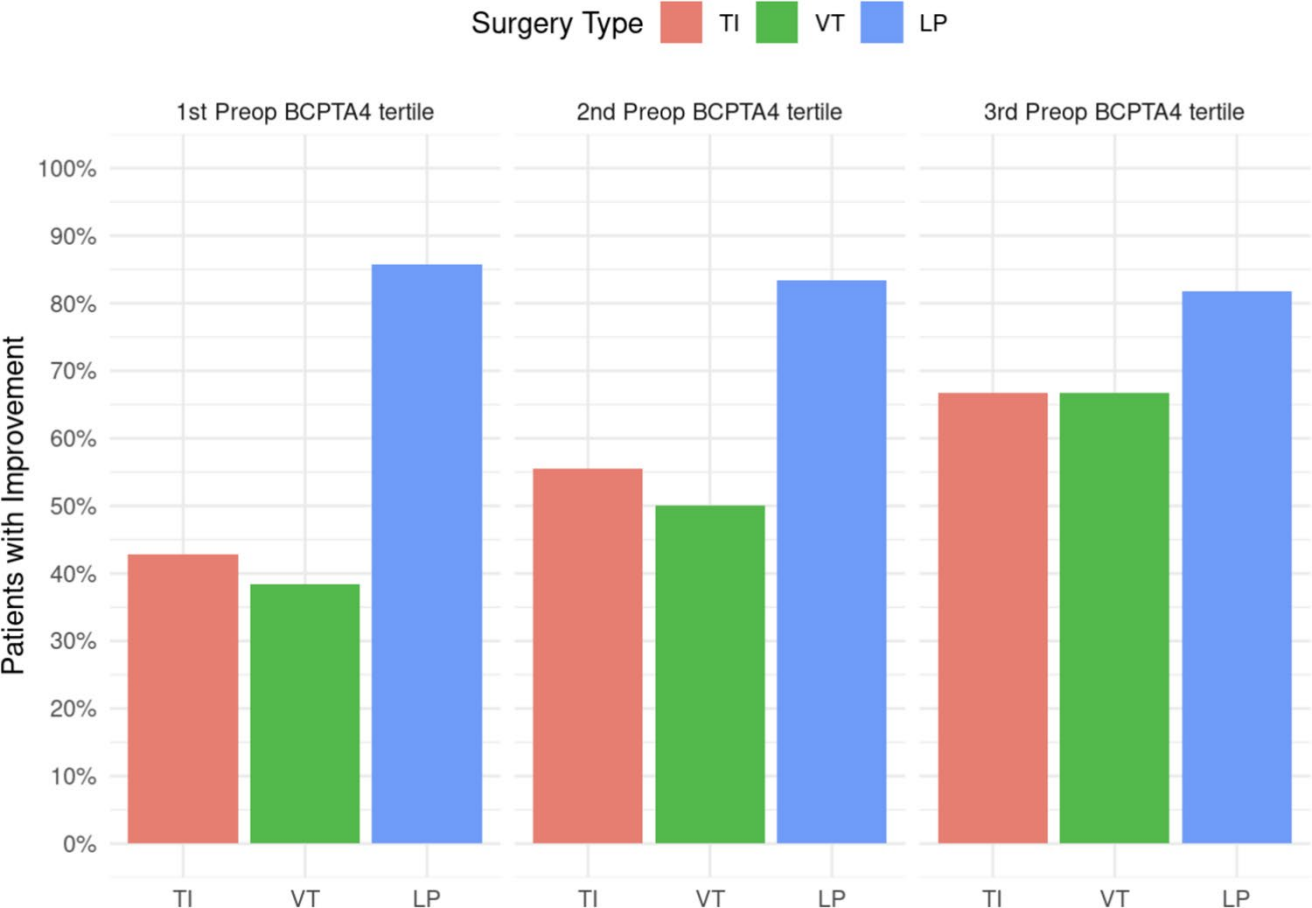
Percentage of patients with improvement in BCPTA4 by therapy modality and subgroup. Bar plot of the proportion of patients with post-therapeutic improvement of PC-PTA4 in all three subgroups, depending on the therapy modality used

With regard to the therapy modality used, there were relevant differences in the improvement in hearing thresholds achieved in the three subgroups *(*Fig. 5*)*. While the TI and PR group showed a relative improvement in BCPTA4 only in the second and third subgroup, the LP group showed a relevant and uniform relative improvement in BCPTA4 in all subgroups.

Regarding time to closure of tympanic membrane perforation, no significant difference was found between the three subgroups (*p* = 0.8). In the tertile subgroups no significant differences were found with regard to the number of follow-up interventions required due to complications (*p* = 0.3).

## Discussion

Despite the limited number of studies, glucocorticoids are well-established in the treatment of idiopathic sudden sensorineural hearing loss (SSNHL) [14, 26]. Intratympanic corticosteroid injections provide benefits over systemic administration, including higher perilymphatic corticosteroid concentrations and fewer systemic side effects [16, 23].

However, a standardized treatment regimen for idiopathic SSNHL remains absent, leading to variations in treatment approaches [24].

Variations exist not only in the active substances, treatment duration, and intervals between applications but also in the methods of administration, leading to potential differences in patient outcomes.

This study compared three intratympanic corticosteroid administration methods: (1) transtympanic injection (TI) under local anesthesia, (2) laser paracentesis (LP) under general anesthesia, and (3) ventilation tube insertion (VT) under general anesthesia.

### Pain

A significant majority of patients (91.9%) reported no pain during treatment. The LP group had the lowest incidence of pain (5.4%), though differences between groups were not statistically significant.

Pain management varies across methods, with TI potentially causing more variable pain due to local anesthesia. In contrast, the general anesthesia used for VT and LP ensures better analgesia during the procedure, which may enhance patient comfort and compliance.

Subsequent instillations in the VT and LP groups are conducted through the existing tympanic membrane perforation, minimizing additional invasiveness and associated discomfort. In contrast, follow-up injections in the TI group typically necessitate re-establishing or locating the puncture site under local anesthesia, which may lead to increased discomfort. Effective pain management, particularly as achieved with LP, is crucial for patient adherence and the overall success of the treatment. Pain during and after the injection can lead to therapy discontinuation or reduced adherence to behavioral measures essential for optimal drug absorption through the round window membrane [16].

In line with this reasoning, we believe that generous analgesia, such as that used in laser paracentesis therapy under general anaesthesia, is appropriate.

### Time to closure and complications

The time to tympanic membrane closure differed significantly among groups. The PR group had the longest time to closure (median (IQR): 90 (90, 90) days), compared to the LP (median (IQR): 30 (22, 41) days) and TI groups (median (IQR): 19 (11, 29) days).

The requirement of a follow-up surgery due to encountered complications also differed significantly between the respective treatment modalities. The four cases in which revision surgery was necessary due to persistent post-therapeutic tympanic membrane perforation belonged to the PR group.

Contrary to otitis media with effusion, paracentesis in the context of ICI is not necessary for middle ear drainage but for corticosteroid administration. Given the short duration of the treatment regimen (three days), prolonged tympanic membrane perforation is unnecessary and undesirable due to risks of middle ear infections or cholesteatoma [27, 28]. A persistent conductive hearing loss can subjectively simulate inadequate therapeutic success for the patient and thus act as a possible stress factor for further recovery.

Therefore, due to their shorter closure times and significantly fewer follow-up surgeries, the LP and TI methods may be more suitable for patients undergoing short-term intratympanic corticosteroid therapy compared to the VT method.

### Hearing threshold

In our study cohort a significant improvement of BCPTA4 was achieved under therapy. The VT and LP groups both showed significant improvements, with the LP demonstrating a greater effect size than the VT group. In contrast, the TI group showed a statistically non-significant improvement in hearing threshold.

Additionally the evaluation of the respective subgroups showed a more consistent relative improvement in BCPTA4 in the LP group, compared to the VT and TI group, suggesting its effectiveness independent to the pretherapeutic BCPTA4.

In the absence of significant differences in pre-treatment, preoperative BCPTA4 and elapsed time between pre-treatment and ICI, three factors should be discussed with regard to the differences in efficacy depending on the form of application.

### Pre-existing conditions

Pre-existing conditions were not accounted for in this study. VT and LP require general anesthesia, which necessitates thorough medical clearance and may exclude patients with significant pre-existing conditions. In contrast, TI is performed under local anesthesia, making it feasible for patients with varying medical statuses. As such, patients with severe conditions may be more likely to receive TI, potentially affecting the results and skewing comparisons.

### Inpatient vs. outpatient setting

Another significant difference between the VT, LP and TI groups is the treatment setting. VT and LP, requiring general anesthesia, are generally performed in an inpatient setting. In contrast, TI is typically conducted on an outpatient basis. Outpatient treatment may involve stressors such as travel, waiting times, and discomfort during or after injection. Inpatient care often mitigates these stressors with immediate medical support, potential bed rest, and reduced procedural discomfort due to general anesthesia.

As demonstrated by Ajduk et al. in their single-center, retrospective study, patients with SSNHL exhibited significantly less improvement in hearing thresholds when experiencing high stress levels during steroid treatment [29]. Administering local glucocorticoids in an inpatient setting, typical of LP and VT procedures, could potentially mitigate these elevated stress levels and thereby enhance treatment effectiveness.

### Exposure to the round window membrane

As demonstrated by Chandrasekhar and co-authors. in their animal model, intratympanic cortisone injection results in significantly higher corticosteroid levels in the periplymph compared to systemic administration [16]. The transport of the active substance in intratympanic corticosteroid injections (ICI) occurs through the three-layered round fenestrated membrane via pinocytosis.

Goycoolea and Lundman postulate that the permeability of the round window membrane depends, in part, on the concentration of the substance reaching the membrane [30]. Therefore, maximizing the duration of corticosteroid interaction with the round window membrane seems crucial in ICI.

In ICI procedures, appropriate patient positioning and behavioural measures can extend the exposure time. These measures are typically easier to implement in the context of injections performed under general anesthesia. In the perioperative setting, appropriate positioning in the horizontal plane is assured, the Eustachian tube remains closed due to the intraoperatively secured airway, and there is an absence of swallowing. Consequently, injections performed under general anesthesia likely result in a longer exposure time of the corticosteroid to the round window membrane, thereby leading to higher corticosteroid levels in the perilymph and thus explaining the superior efficacy observed with the respective administration techniques.

## Conclusion

Both intratympanic cortisone injections, performed by laser paracentesis or by insertion of a ventilation tube demonstrated significant improvement in hearing thresholds among patients with sudden sensorineural hearing loss (SSNHL). However, based on the greater effect size observed in our study, the consistent effectiveness independent on the severity of the SSNHL and the short time to closure of tympanic membrane perforation, laser paracentesis emerges as the preferred method for administering intratympanic glucocorticoids in the management of SSNHL.

The findings underscore the potential advantages of laser paracentesis in achieving better therapeutic outcomes, likely due to optimized corticosteroid delivery and faster closure of the tympanic membrane perforation. Future prospective studies should further investigate the differences and long-term efficacy of these administration techniques in order to refine treatment protocols for SSNHL.

## Data Availability

The datasets analysed during the current study are available from the corresponding author on reasonable request.

## Declarations

This article does not include any human or animal studies conducted by the author. All patients who can be identified via images or other information within the manuscript have given their written consent.
